# *In vitro* comparison of the accuracy of four intraoral scanners and three conventional impression methods for two neighboring implants

**DOI:** 10.1371/journal.pone.0228266

**Published:** 2020-02-27

**Authors:** Elena Roig, Luis Carlos Garza, Natalia Álvarez-Maldonado, Paulo Maia, Santiago Costa, Miguel Roig, José Espona

**Affiliations:** 1 Department of Restorative Dentistry, Universitat Internacional de Catalunya, Barcelona, Spain; 2 Instituto Superior de Ciências da Saúde - Egas Moniz, Caparica, Portugal; Eberhard-Karls-Universitat Tubingen Medizinische Fakultat, GERMANY

## Abstract

**Purpose:**

To determine whether the accuracy of two-implant model impressions taken with optical scanners was inferior to that of those taken with elastomeric materials.

**Materials and Methods:**

Impressions of a resin reference model with two almost parallel implants were taken using three elastomeric impressions (closed tray technique, open tray nonsplinted technique and open tray splinted technique) and scanned with four optical scanners (CEREC Omnicam, 3M True Definition Scanner, 3Shape TRIOS3 and Carestream CS 3600). STL files of the different methods were superimposed and analyzed with control software (Geomagic Control X, 3D systems) to determine the mean deviation between scans.

**Results:**

Compared to elastomeric impressions, optical impressions showed a significantly improved mean precision. TRIOS3 and CS3600 showed a significantly improved mean trueness compared to that of closed tray, CEREC Omnicam and TrueDefinition. All methods showed a certain degree of implant rotation over their axes, which was significantly higher in the closed tray and the open tray nonsplinted techniques.

**Conclusions:**

Optical impressions, taken under these in vitro conditions, showed improved accuracy compared with that of elastomeric impressions.

## Introduction

Accuracy is crucial to the true passive fit of implant prostheses[1], which the existing clinical procedures and laboratory fabrication methods are unable to achieve. Without a true passive fit, also called misfit, the stresses in the implanted prostheses are directly transferred to the mechanical components and surrounding bone[2]. Misfit may lead to bacterial microleakage, screw loosening or component stress and fracture[3–5].

Taking impressions using elastomeric materials to capture the position of the dental implant has become the most widely used technique and remains the gold standard. However, the elastomeric method has procedural shortcomings, and this technique is uncomfortable for the patient and inconvenient for the clinician[6–8].

To address these downsides and to maintain or improve the accuracy of elastomeric methods, several new optical impression systems have been introduced to the market[9]. These systems appear to improve patient experience[10–12] and reduce material costs and time[11, 13]. Some authors believe these optical impression systems have minimal distortion, which confers adequate clinical longevity to the prosthesis due to acceptably associated stress[14]; but these systems are, at present, unable to achieve a true “passive” fit. However, when used for short bridges or crowns, these systems fulfil the minimum requirements of accuracy[15]. A number of studies have reported that the inaccuracies associated with the systems used for implant impressions are too significant to be acceptable[16]. Although some studies claim that these systems provide sufficient accuracy in complete-arch impressions, scientific evidence on the intraoral scanning of complete-arches with teeth is lacking and outdated[17]. Elastomeric impressions of complete arches are significantly more accurate than those of optical arches[18] and the precision of intraoral scanners decreases as the distance between each scan body increases[19–21]. However, when only two implants are scanned, the accuracy of IOS improves[22]. In the case of IOS, in contrast to conventional impressions, the angulation of the implants does not affect the accuracy [23]. Digital systems have gained wider acceptance in dentistry due to the emergence of more user-friendly and more accurate systems.

The verdict on digital impression accuracy remains inconclusive, and direct comparisons between implant impressions and digital alternatives are needed[24]. The present study aims to compare the accuracy of optical impressions recorded by several intraoral scanners with the accuracy of conventional impressions using elastomeric materials over implants in a partially edentulous model. To this end, we selected four optical systems: TrueDefinition (3M, USA), TRIOS3 (3Shape, Copenhagen, Denmark), CEREC Omnicam (Dentsply-Sirona, Bensheim, Germany), and CS3600 (Carestream, Atlanta, USA). The null hypothesis of the present study was that optical intraoral impressions were less accurate than conventional implant impressions were.

According to ISO 5725[24], the term “accuracy” refers to both trueness and precision. “Trueness” denotes the closeness of agreement between the arithmetic mean of a large number of test results and the “true” or accepted reference value. “Precision”, referring to the closeness of agreement between test results, is normally expressed in terms of standard deviations. To evaluate accuracy, both trueness and precision must be assessed.

The clinically acceptable degree of inaccuracy is difficult to determine because even minimal discrepancies seem to cause significant stress in the framework[4]. Some authors consider 30 μm to be acceptable[25], while other studies have proposed a limit of 150 μm to avoid long-term prosthetic problems[26].

However, the purpose of this study was not to determine the acceptable degree of inaccuracy but to establish whether optical impression systems were inferior to conventional impression systems in a two-implant model.

## Materials and methods

**Fig 1:**
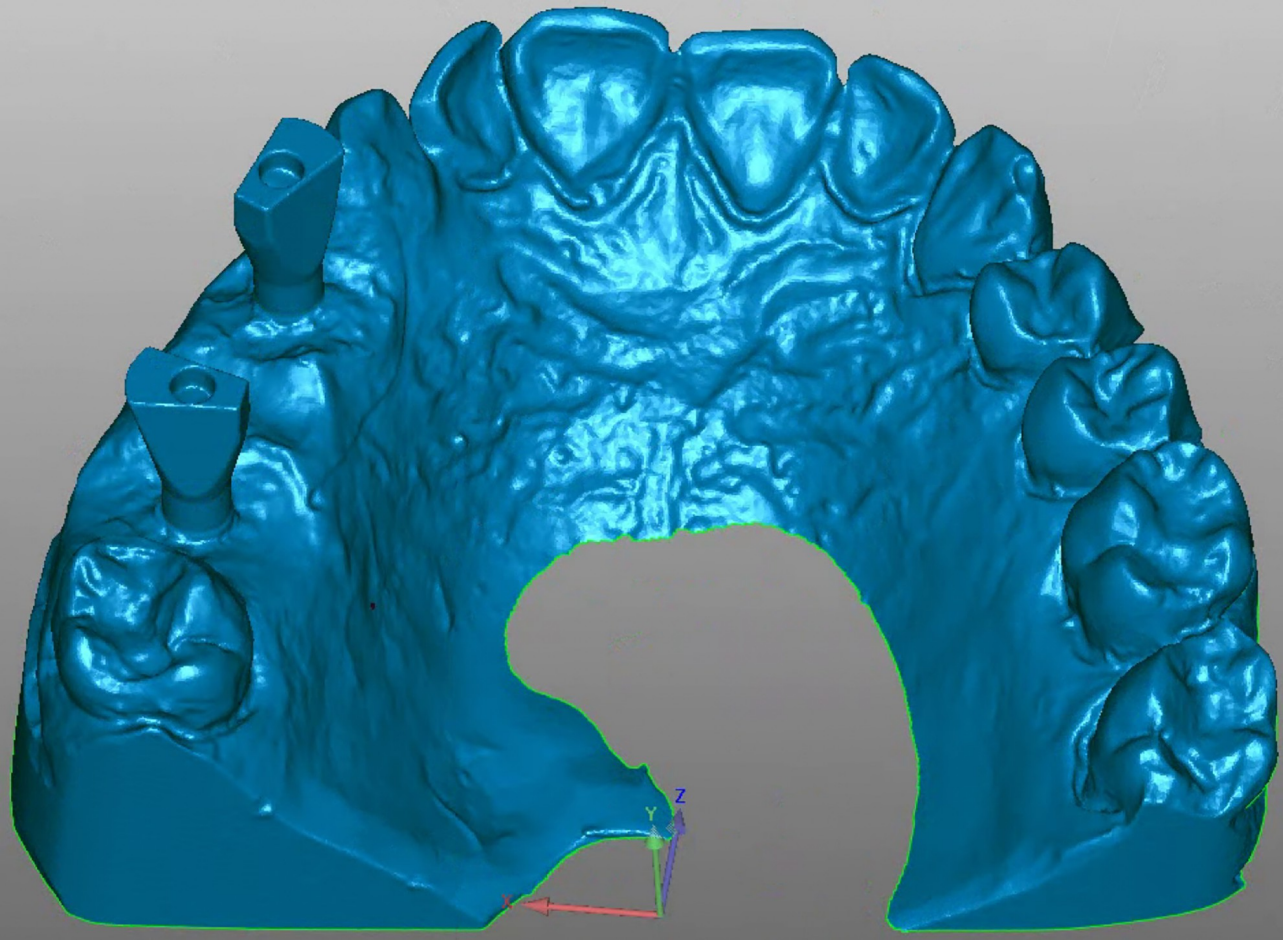
STL model obtained by scanning the model with the scan bodies screwed onto the implant analogs.

**Fig 2:**
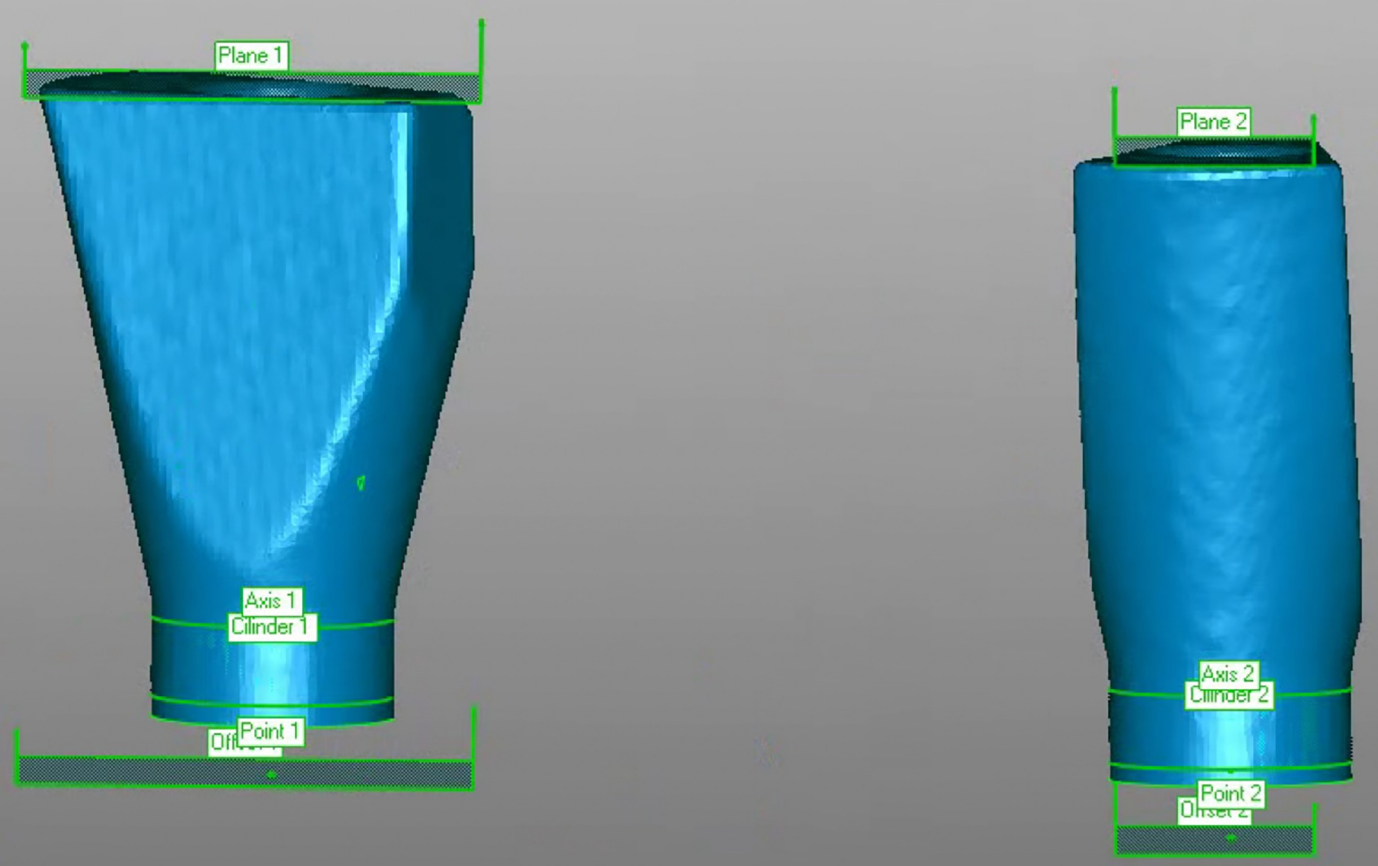
A plane was constructed on top of the scan body (plane 1). An offset plane was obtained by a -10 mm reduction apically (offset 1). A cylinder was constructed based on the shape and the axis (axis 1) of the scan body (cylinder 1). The intersection of offset 1 and axis 1 was considered the center of the implant head, or centroid (Point 1).

Epoxy resin was used to fabricate a master model missing teeth 1.4 to 1.6 restored with two internal connection implants at an almost parallel configuration (C1 MIS Implants, MIS‐Implants Inc., Shlomi, Israel) in positions 1.4 and 1.6. A scan body (Scan Post CS-SP102, MIS implants) was screwed onto each implant, and the model was scanned three times with a desktop scanner (3Shape D810; 3Shape, Copenhagen, Denmark) (Fig 1). Three stereolithographic (STL) files obtained from the scanner were imported into Geomagic Control X (3D Systems Inc., Rock Hill, SC, USA) and aligned by pairs using the best fit method. The axis of the scan body was established, after which a plane was constructed on its coronal flat surface (plane 1) and then moved 10 mm apically (offset 1). The intersection between the offset and the scan body axis was identified as the center of the implant analog head, or centroid (point 1) (Fig 2). The differences between the centroids in each STL file were measured. The STL file with the least differences was selected as the STL reference file.

Impressions of the model were taken using 4 intraoral optical scanners and 3 conventional impression techniques, and ten impressions were fabricated for each group.

### Optical intraoral scanners

The scan bodies were screwed into the implants position closest to that in the reference model, with a 10 Ncm screwing torque. Two calibrated operators used each scanner to take five optical impressions of the model, and these scans were exported as STL files. The scanning protocol was started at the second right molar, the tooth distal to the distal implant, and subsequently the scanner was swept over the occlusal surface up to the first left molar. Returning to the second right molar, the operator rolled and wiggled the scanner to capture the buccal-palatal surfaces up to the second left molar. The following groups were studied:

1. **Group CS**. CS3600 3.1 (Carestream, USA)
2. **Group TR**. TRIOS3 1.18.2.10 (3Shape TRIOS, Denmark)
3. **Group OC**. CEREC Omnicam SW4.6.1 (Dentsply Sirona, Germany)
4. **Group TD**. TrueDefinition L51 V01.33 (3M True Definition, Germany). To facilitate scanning with this scanner, powder (3M High resolution scanning spray, 3M, Germany) was first sprayed onto the model surface.

### Conventional impressions

1. **Group CT**—closed tray impression. After placing two closed tray impression copings (CS IC485, MIS implants) onto the dental implants of the master model, a polyether halfway(Impregum Penta; 3M ESPE, Germany) complete arch impression was taken following the manufacturer’s instructions. Rim-Lock metal trays (Dentsply Sirona, Orange, USA) without polyether adhesive were used. Once the material had set, the impression was removed from the model. Subsequently, the transfer copings were unscrewed from the master model, and the implant analogs were repositioned under 3.8x magnification and good lighting into the transfer copings. One hour later, CAD/CAM type IV stone plaster (Ventura scan stone, Madespa, Spain) was vacuum-mixed, in accordance with the water/powder proportions (20 ml, 100 g) recommended by the manufacturer, and poured into the impression. According to the manufacturer, expansion at 2 h is 0.08%. After 2 h, the impression tray was removed, and the transfer copings were replaced with the scan bodies. Given that each scan body has six possible positions in the implant analog, utmost care was taken to place the two scan bodies in the same position as that in the reference model. Subsequently, the model was scanned using a desktop scanner (D810; 3Shape, Copenhagen, Denmark), and an STL file was obtained.
2. **Group OS**—open tray splinted impression. Two open tray implant impression copings (CS IO485, MIS implants) were placed on the dental implant, where they were splinted and unified with a clear colorless Triad gel light cure material (Dentsply International, York, PA), which was polymerized for at least 60 seconds in each section. After polymerization, the resin structure was cut using a 0.8 diamond disk approximately halfway between the implants. Twenty-four hours later, the structure was resplinted with tiny amounts of the same gel to reduce the shrinkage of the resin. A plastic tray (Impression Tray, 3M ESPE) was perforated with two holes corresponding to the positions of the transfer copings to allow the placement and removal of the screws. An impression was taken with polyether, in accordance with the manufacturer’s instructions. Once the impression material had set, the impression was removed by unscrewing the transfer copings. Implant analogs were then screwed into the transfer copings fixed to the impression. The impression was then poured, as in group CT.
3. **Group ON**—open tray nonsplinted impression. Two open tray transfer impression copings (CS IO485, MIS implants) were screwed into the dental implants. Two perforations were made in a plastic tray (Impression Tray, 3M ESPE) according to the positions of the transfer copings to allow the placement and removal of the screws. The impressions were then taken and poured, as in the OS group.

Two calibrated operators took the impressions using the scanners that they had been trained to use following the same scanning protocol. As differences between operators have been shown, operators completed a one-hour session on how to take elastomeric impressions[27].

Different measurements were taken to assess accuracy:

### 3D displacement of the centroids

Geomagic Control X was used to superimpose the STL test files over the STL reference files. The STL scan bodies were then aligned using the reference alignment and the best fit alignment and exported as a single file.

**Fig 3:**
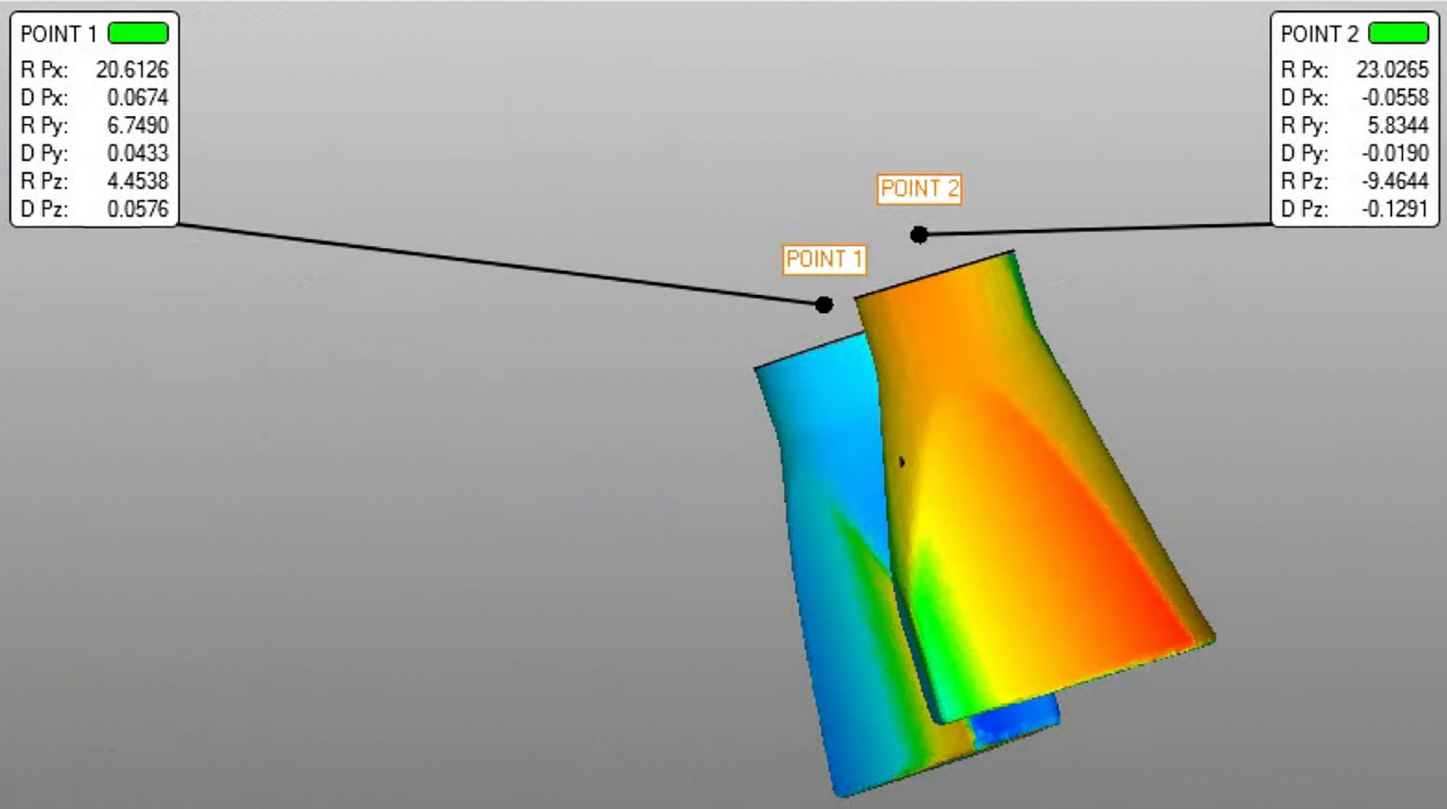
The best fit alignment was used to measure the distance between the two points.

After determining a point at the center of each implant head, also called the centroid[28], each scan body axis was established. This procedure provided data on the three-dimensional axes (x, y and z-axes) as the coordinate values that are transformed into linear and angular data. Then, the distances between the reference files and the test centroids were analyzed (Fig 3). The reference and best fit superimposition methods were used. In the reference method, the test STL and the reference STL were aligned with the first implant using a scan body, while for the best fit method, all the scan bodies were aligned with the implants at the same time. The best fit method distributes the differences among all implants, while the reference method shows the maximum possible differences.

### Distance between the two implant centers

**Fig 4:**
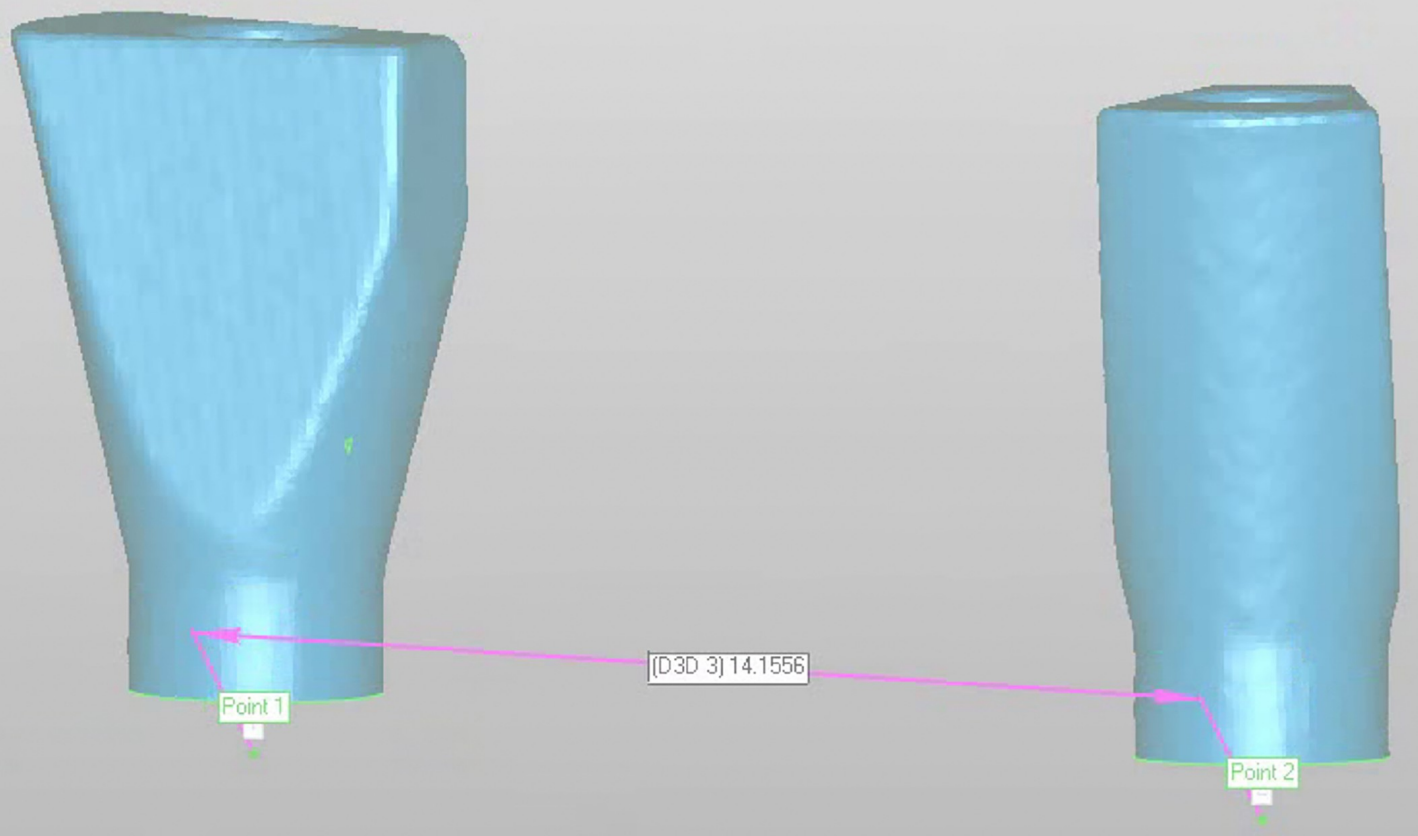
Measuring the distance between the centroids of the two implant heads.

The distance between the centers of each implant head was measured and subtracted from the distance in the reference (Fig 4).

### Rotation of the implants over their axes

**Fig 5:**
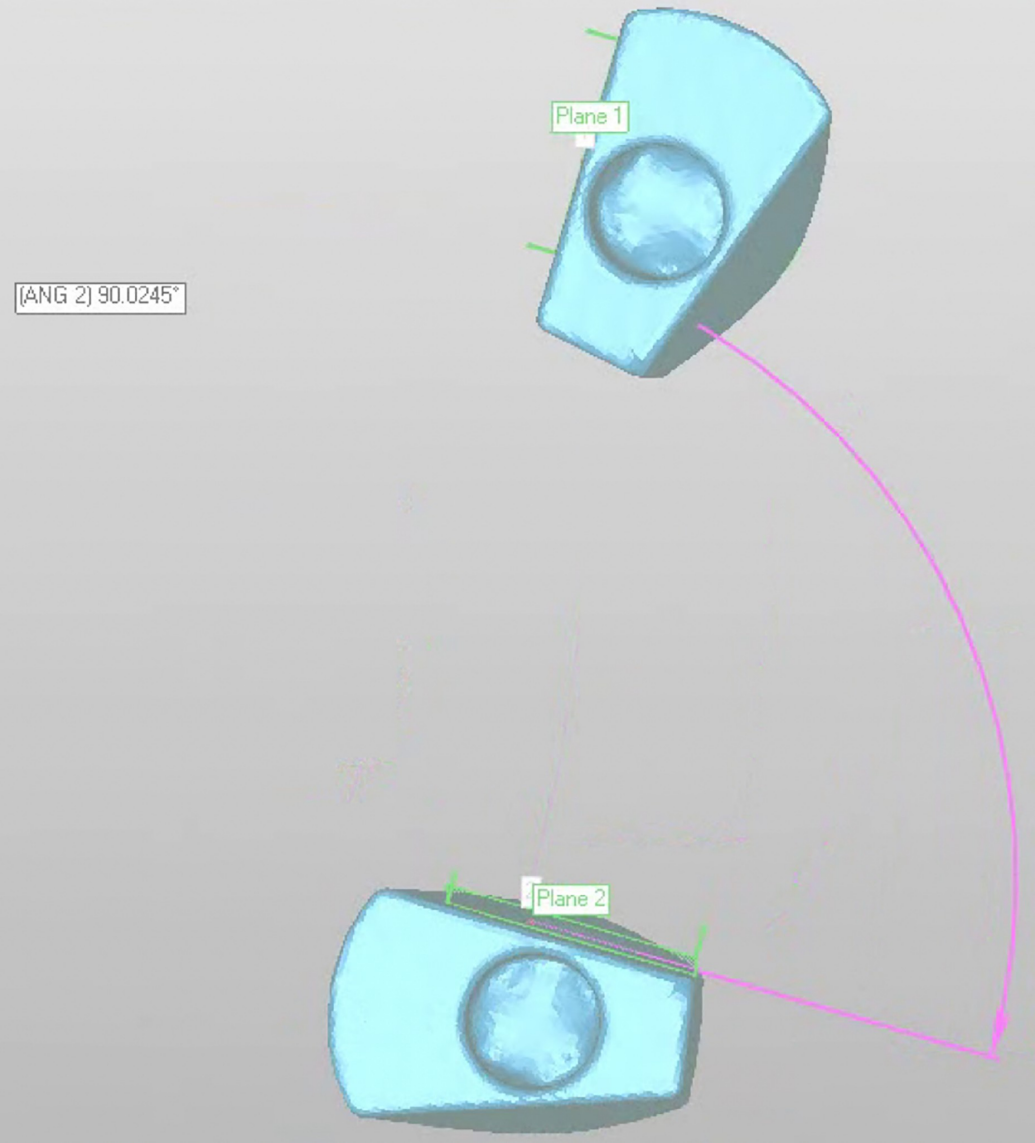
Two planes were constructed on a wall parallel to the implant axis of each scan body, and the angle between them was determined.

After constructing a plane on a wall parallel to the axis of each scan body, the angle between the two planes was determined (Fig 5). The deviation was then calculated by subtracting the angle of the reference model.

### Precision

Precision was analyzed by comparing each set of STL files with all STL files taken with the same scanning system. The root mean square (RMS) error obtained was used to assess precision[29].

Levene’s test and the Shapiro-Wilk test (p<0.05) were used to determine normality of variance and distribution. One-way analysis of variance (ANOVA) with Fisher’s least significant difference (LSD) post hoc test was used to compare means between groups (p<0.05). Statgraphics centurion XVII software (Statgraphics Technologies, Virginia, USA) was used to analyze the results.

## Results

### 3D displacement of the centroids

Significant differences (p<0.05) were observed with one-way analysis of variance (ANOVA). As significant differences were found (p<0.05), the LSD post hoc test was used to identify homogeneous groups. Group means were compared in pairs to ensure homogeneity (Table 1). The results of Carestream 3600 and TRIOS3 were significantly inferior to those of the closed tray technique, open tray technique, CEREC Omnicam and True Definition scanning systems.

**Table 1:** Comparison of the mean distance between each implant head center in the STL test file and the STL reference file. As significant differences were found (p<0.05), the LSD post hoc test was used to identify homogeneous groups.

	POINT 1		POINT 2	
SYSTEM	Mean (mm)	Homogeneous groups	Mean (mm)	Homogeneous groups
CS3600	0.012	X	0.018	X
Master model	0.018	X X	0.020	X
TRIOS3	0.019	X	0.024	X X
Closed tray	0.034	X	0.047	X
Open tray non-splinted	0.047	X	0.056	X
Open tray splinted	0.059	X	0.060	X X
CEREC Omnicam	0.225	X	0.063	X X
TrueDefinition	0.235	X	0.078	X

Method: 95.0 percent LSD. Within each column, the levels containing X’s for a group of means within there are not statistically significant differences.

### Distance between the two implant centers

**Fig 6:**
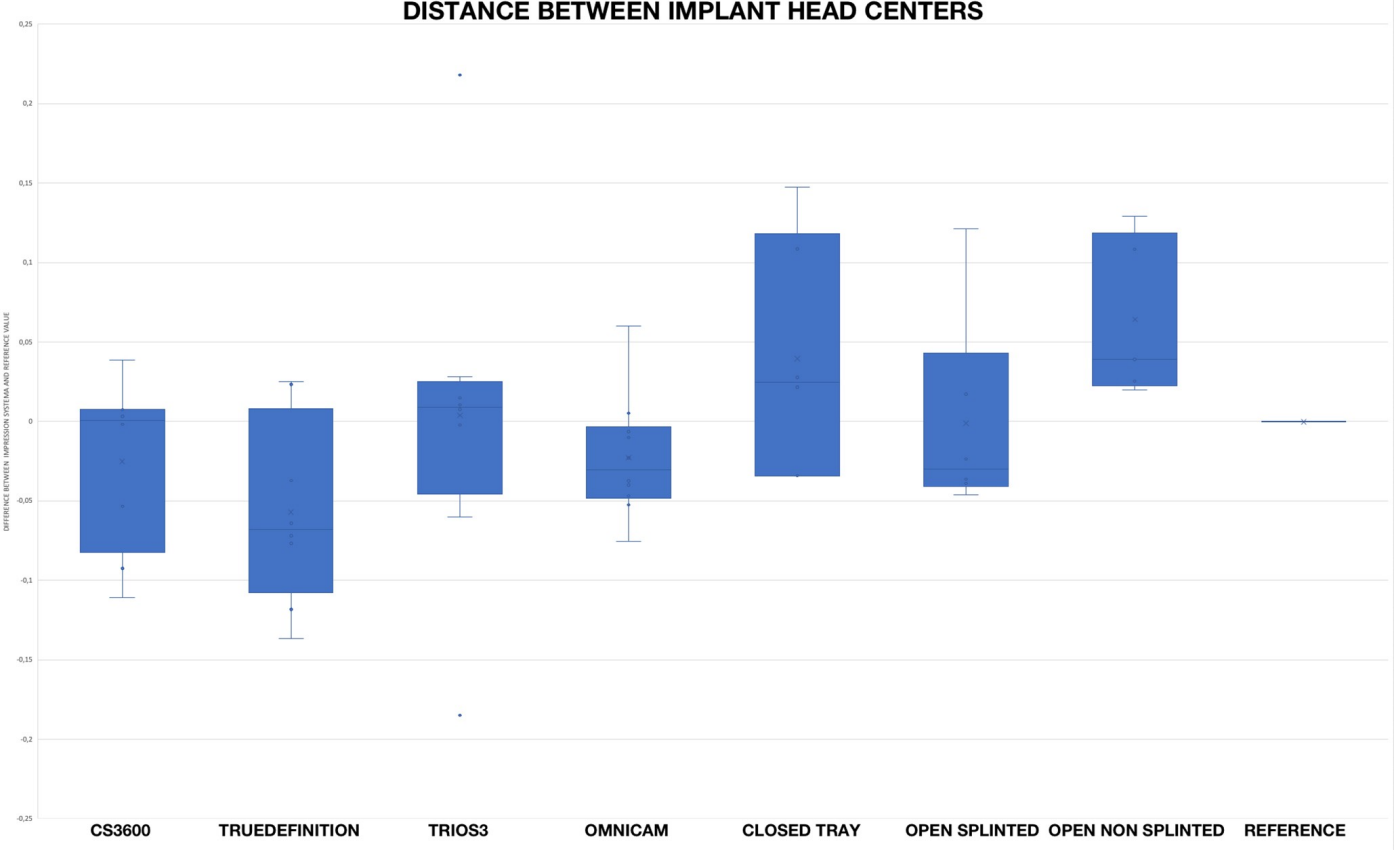
Differences between the distance of the two centroids of the test model and the reference model.

Fig 6 shows the distances between the two centroids of the test model and the reference model. The distances of the optical impression groups did not appear to be inferior to those of the conventional groups.

### Rotation of the implants over their axes

**Fig 7:**
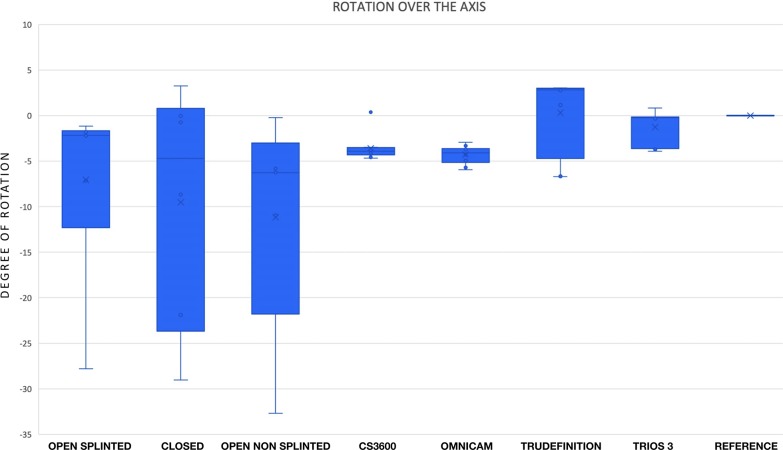
The differences in the angle between the two flat horizontal surfaces of the two implants in the test and the reference models.

All systems used showed a certain degree of rotation. The differences in the angle between the two flat horizontal surfaces of the two implants in the test and the reference models are shown in Fig 7. The nonsplinted elastomeric impressions revealed significantly inferior results than those of the optical impressions. No significant differences were found between the open splinted elastomeric impressions and any of the other 6 impression systems analyzed or between the closed impression and any of the other six impression systems (Table 2).

**Table 2:** Comparison of the implant rotation over their axes for each group.

Multiple Range Test for Angle by System		
System	Mean (degree of rotation)	Homogeneous groups
Open tray non-splinted	86.040	X
CEREC Omnicam	87.568	X X
CS3600	88.259	X X X
Open tray splinted	88.939	X X X X
Closed tray	90.296	X X X
TRIOS3	90.579	X X
TrueDefinition	92.153	X

Method: 95.0 percent LSD. Within each column, the levels containing X’s for a group of means within there are not statistically significant differences.

### Precision

**Fig 8:**
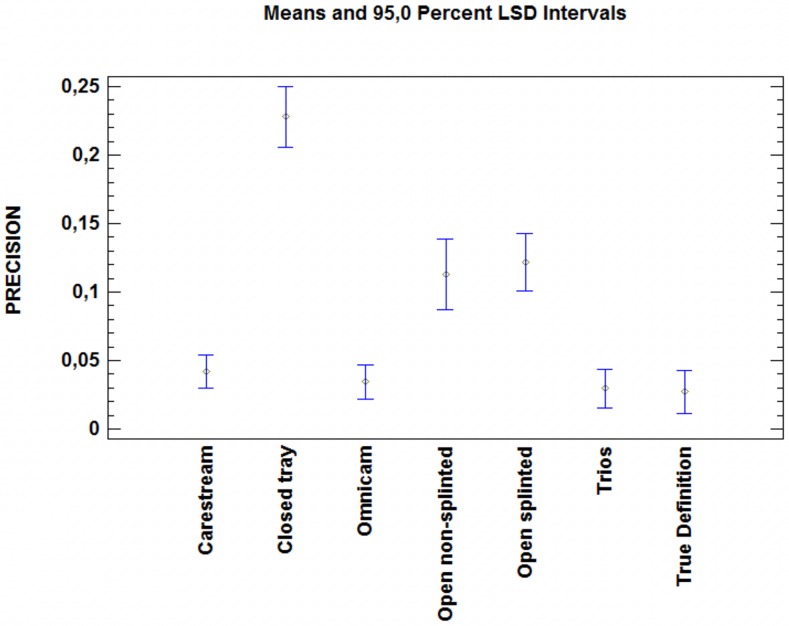
One-way ANOVA comparing the precision of the methods analyzed. The optical methods showed significantly more precision than the elastomeric methods did. The open tray impressions were significantly more precise than the closed tray impressions were (p<0.05).

No significant differences were observed between the optical impressions. In addition, these impressions were significantly more precise than the elastomeric impressions (p<0.05) (Table 3). No significant difference was found between the two open tray methods, although both methods were significantly more precise than the closed tray method (p<0.05) (Fig 8).

**Table 3:** Comparison of the precision among systems.

Multiple Range Test for Precision by System		
System	Mean	Homogeneous group
TrueDefinition	0.027	X
TRIOS3	0.029	X
CEREC Omnicam	0.034	X
CS3600	0.042	X
Open tray non-splinted	0.113	X
Open tray splinted	0.121	X
Closed tray	0.227	X

Method: 95.0 percent LSD. Within each column, the levels containing X’s for a group of means within there are not statistically significant differences.

## Discussion

Two samples (CEREC Omnicam and CS3600) were discarded because they could not be aligned with less than 20 μm of misfit, despite the calibration of the operators. Both discarded files revealed evident defects in the impressions. Following the same protocol as that for conventional impressions, the clinician must carefully check optical impressions for defects before delivering them to the technician. If a defect is identified, then a new optical impression must be taken.

Regarding the precision of conventional impressions, in the conditions of our in vitro study, conventional impressions are significantly less precise than optical impressions are. It is important to highlight the high variability in different studies on linear and 3D distortion values, which range between 2 μm and 180 μm [30–33]. According to Baig [34], there is currently no evidence to support the splinting of impression copings to improve implant impression accuracy. Nevertheless, our results with respect to conventional impressions are similar to those of Izadi et al., who also found that open tray impressions were better than closed tray impressions [35]. The type of implant used might also contribute to differences in accuracy. Osman et al. also concluded that open tray impressions were more accurate than closed tray impressions, although in some implants, there was no difference [23]. Osman et al showed that the accuracy values were low, but these authors only measured the horizontal discrepancy in micrometers, whereas in the case of vertical discrepancy, a qualitative assessment of the presence or absence of discrepancy was performed. In our case, overall 3D discrepancy was measured[23]. Additionally, the type of gypsum might explain the differences between the studies, although some authors consider that the type of gypsum used is not important[36], while other authors claim better accuracy for certain types of gypsum [37]. The morphology and length of the impression copying can also determine differences between different studies[38].

Both the test and the reference STL files were aligned using Geomagic reference fit and best fit options. The reference fit option superimposes the first scan body and then calculates the difference between the centroid of the first and the second scan body[39]. Nevertheless, as superimposition is never perfect[39], the error is magnified in the subsequent scan body. Therefore, we discarded the models based on the first implant references and used the best fit option, which aligns the two scan bodies simultaneously.

When screwing the bridge on several implants, the clinician never screws on each implant individually but alternates between the implants. Once all screw joints have been tightened, the final torque is applied. This procedure compensates for any inaccuracies. The final result we obtained was the maximum difference in every implant, instead of the increasing difference in every next implant.

As the impression-taking process in all the groups took almost two months, we had to scan the master model every week to ensure its stability and to check whether any possible variations in the position and rotation of the implants occurred in the master model. A mean deviation below 6 μm indicated that the model was stable and that there were no changes in the implant position over time[40].

According to our results, under in vitro conditions, optical scanners are not inferior to conventional techniques for taking impressions of two almost parallel implants between teeth. Nevertheless, the results of the present study do not necessarily correspond to the clinical results. In the case of optical impressions, the presence of humidity and the mobility of the soft tissues surrounding the scan bodies can significantly affect the scanning process and the impression accuracy. In the case of elastomeric impressions, humidity can also alter the accuracy of the results. Closed tray impressions were significantly less accurate in terms of 3D displacement than were splinted open tray impressions. No significant difference was found between closed tray and nonsplinted open tray impressions or between splinted and nonsplinted open tray impressions.

Given that some studies have claimed polyether to be more accurate than polyvinyl siloxane impression material, we chose polyether for conventional impressions[41]. Knowing that time can affect impression accuracy, we waited one hour before pouring the impression[7]. Water to powder proportions were followed according to the manufacturer’s instructions. Although some authors have claimed that conventional impressions are more accurate than optical impressions for two consecutive implants[16], our results did not show an inferior performance of the optical impression techniques when compared to conventional impressions. These findings could be because the many steps involved (impression making stages, master cast, resin verification jig, waxing, investing, casting, veneer addition and finishing) can distort the final outcome[1]. The optical devices yielded a result in the range of 50–60 μm, suggesting these devices could be used for clinical impressions.

No significant difference was found between splinted and nonsplinted open tray impressions in the present study. This finding is in accordance with studies claiming that when highly rigid impression material (such as polyether) is used, the splinting of pick-up impression copings with acrylic resin is not useful to improve precision[42].

One possible issue regarding precision is the rotation of the implant analog, which might clinically affect the model. Implant analog rotation over the axis was determined by the angle between the two vertical flat surfaces of the scan body. Unlike elastomeric impressions, optical impressions appeared to reduce the risk of implant analog rotation. However, elastomeric impressions with splinted abutments rotated less than the nonsplinted abutments. When splinted frameworks with nonengaging connections are required, no rotation occurs, but the use of engaging connections might compromise the clinical result. In open tray impressions, extreme care was taken when placing the implant analogs in the impression transfer copings. All procedures were performed under magnification and good lighting.

The implants used in the model were placed almost parallel to each other, and the distance between them was the ideal for placing a molar and a premolar on top with a premolar pontic between them. According to Chia et al.[43], placing the implants angulated would probably lead to worse results for conventional impressions, while optical results would have probably been less affected. The distance between the implants was relatively wide (one pontic in between), which is not the best scenario for implant impressions[22, 44], but this scenario does not seem to affect conventional impressions[21]. Nevertheless, the results for the optical impressions did not seem to be affected.

A possible limitation of this study is the use of a desktop scanner to evaluate conventional models because it is not as accurate as a probe[22]. Nevertheless, we preferred the use of a desktop scanner because it is still highly accurate[38] and, moreover, a desktop scanner is commonly used by lab technicians to capture conventional models to proceed with their prosthodontic designs.

Another possible limitation is the continuous changes in the device software. Although accuracy should be improved, it could also become worse [22], so continuous assessment of the new software versions is needed.

From a clinical perspective, intraoral scanners have advantages and drawbacks. Patients generally have an overall better perception of IO scanning than of conventional impressions[45]. Optical scanning seems to be a more didactic and preclinical instruction; however, this method requires a rapid increase with multiple practice attempts [46].

## Conclusions

Our findings suggest that optical impressions are superior to elastomeric impressions for placing two implants in one quadrant. Closed tray impression accuracy was significantly lower than that of open tray impressions for placing two implants in one quadrant.

## Supporting information

S1 File Results of the study. All results obtained in the study are listed in this file.
(DOCX)
