# Supplementary material for: In vitro comparison of the accuracy of four intraoral scanners and three conventional impression methods for two neighboring implants
Source: PLoS One. 2020 Feb 27;15(2):e0228266. doi: 10.1371/journal.pone.0228266 (PMC7046187; doi:10.1371/journal.pone.0228266)
Supplement: S1 File — All results obtained in the study are listed in this file. (DOCX) [file pone.0228266.s001.docx]

DISTANCE

| CS01 | CS 3600 | 14,1096 |
| --- | --- | --- |
| CS02 | CS 3600 | 14,2001 |
| CS03 | CS 3600 | 14,1172 |
| CS04 | CS 3600 | 14,2053 |
| CS05 | CS 3600 | 14,2405 |
| CS06 | CS 3600 | 14,2099 |
| CS07 | CS 3600 | 14,2094 |
| CS08 | CS 3600 | 14,0913 |
| CS09 | CS 3600 | 14,0725 |
| CS10 | CS 3600 | 14,1486 |
| TD01 | TRUE DEFINITION | 14,0836 |
| TD02 | TRUE DEFINITION | 14,13 |
| TD03 | TRUE DEFINITION | 14,1648 |
| TD04 | TRUE DEFINITION | 14,1251 |
| TD05 | TRUE DEFINITION | 14,0652 |
| TD06 | TRUE DEFINITION | 14,2272 |
| TD07 | TRUE DEFINITION | 14,1379 |
| TD08 | TRUE DEFINITION | 14,2252 |
| TD09 | TRUE DEFINITION | 14,211 |
| TD10 | TRUE DEFINITION | 14,1221 |
| 3S01 | TRIOS 3 | 14,2302 |
| 3S03 | TRIOS 3 | 14,1419 |
| 3S04 | TRIOS 3 | 14,1998 |
| 3S05 | TRIOS 3 | 14,42 |
| 3S06 | TRIOS 3 | 14,017 |
| 3S07 | TRIOS 3 | 14,2124 |
| 3S08 | TRIOS 3 | 14,1976 |
| 3S09 | TRIOS 3 | 14,2168 |
| 3S10 | TRIOS 3 | 14,2226 |
| 3S11 | TRIOS 3 | 14,2095 |
| OC01 | OMNICAM | 14,1551 |
| OC02 | OMNICAM | 14,1265 |
| OC03 | OMNICAM | 14,1956 |
| OC04 | OMNICAM | 14,1646 |
| OC05 | OMNICAM | 14,1789 |
| OC06 | OMNICAM | 14,1919 |
| OC07 | OMNICAM | 14,162 |
| OC08 | OMNICAM | 14,2619 |
| OC09 | OMNICAM | 14,1495 |
| OC10 | OMNICAM | 14,2072 |
| CC01 | CLOSED | 14,3105 |
| CC02 | CLOSED | 14,1676 |
| CC03 | CLOSED | 14,3494 |
| CC04 | CLOSED | 14,2235 |
| CC05 | CLOSED | 14,2297 |
| CC06 | CLOSED | 14,1678 |
| CC07 | CLOSED | 14,291 |
| CC08 | CLOSED | 14,1566 |
| CC09 | CLOSED | 14,3291 |
| CC10 | CLOSED | 14,2114 |
| AF01 | OPEN SPLINTED | 14,1556 |
| AF02 | OPEN SPLINTED | 14,2191 |
| AF03 | OPEN SPLINTED | 14,1657 |
| AF04 | OPEN SPLINTED | 14,3231 |
| AF05 | OPEN SPLINTED | 14,163 |
| AF06 | OPEN SPLINTED | 14,1783 |
| AF07 | OPEN SPLINTED | 14,1566 |
| AF08 | OPEN SPLINTED | 14,1991 |
| AF09 | OPEN SPLINTED | 14,1557 |
| AF10 | OPEN SPLINTED | 14,2236 |
| AN01 | OPEN NON SPLINTED | 14,2273 |
| AN02 | OPEN NON SPLINTED | 14,2218 |
| AN03 | OPEN NON SPLINTED | 14,3103 |
| AN04 | OPEN NON SPLINTED | 14,2411 |
| AN05 | OPEN NON SPLINTED | 14,2187 |
| AN06 | OPEN NON SPLINTED | 14,2221 |
| AN07 | OPEN NON SPLINTED | 14,3004 |
| AN08 | OPEN NON SPLINTED | 14,2398 |
| AN09 | OPEN NON SPLINTED | 14,2291 |
| AN10 | OPEN NON SPLINTED | 14,3309 |
|  | REFERENCE | 14,2015 |
|  | REFERENCE | 14,202 |
|  | REFERENCE | 14,2086 |
|  | REFERENCE | 14,2469 |
|  | REFERENCE | 14,3636 |

ANGLE

| 2S1 | OPEN SPLINTED | 90,0295 |
| --- | --- | --- |
| 2S2 | OPEN SPLINTED | 64,0558 |
| 2S3 | OPEN SPLINTED | 89,6108 |
| 2S4 | OPEN SPLINTED | 89,7045 |
| 2S5 | OPEN SPLINTED | 84,6873 |
| 2S6 | OPEN SPLINTED | 90,667 |
| 2S7 | OPEN SPLINTED | 90,0292 |
| 2S8 | OPEN SPLINTED | 86,0498 |
| 2S9 | OPEN SPLINTED | 89,6219 |
| 2S10 | OPEN SPLINTED | 89,7056 |
| 2C1 | CLOSED | 91,1038 |
| 2C2 | CLOSED | 69,9374 |
| 2C3 | CLOSED | 62,8026 |
| 2C4 | CLOSED | 95,0997 |
| 2C5 | CLOSED | 83,1751 |
| 2C6 | CLOSED | 90,8261 |
| 2C7 | CLOSED | 91,0102 |
| 2C8 | CLOSED | 71,9344 |
| 2C9 | CLOSED | 65,8221 |
| 2C10 | CLOSED | 93,0111 |
| 2NS1 | OPEN NON SPLINTED | 59,169 |
| 2NS2 | OPEN NON SPLINTED | 91,6424 |
| 2NS3 | OPEN NON SPLINTED | 85,5868 |
| 2NS5 | OPEN NON SPLINTED | 86,0355 |
| 2NS6 | OPEN NON SPLINTED | 80,8951 |
| 2NS7 | OPEN NON SPLINTED | 79,187 |
| 2NS8 | OPEN NON SPLINTED | 92,6314 |
| 2NS9 | OPEN NON SPLINTED | 85,6081 |
| 2NS10 | OPEN NON SPLINTED | 86,0295 |
| cs01 | CS 3600 | 87,6987 |
| cs02 | CS 3600 | 88,0664 |
| cs03 | CS 3600 | 87,8374 |
| cs04 | CS 3600 | 87,2788 |
| cs05 | CS 3600 | 87,1893 |
| cs06 | CS 3600 | 88,0133 |
| cs07 | CS 3600 | 87,6091 |
| cs08 | CS 3600 | 88,3662 |
| cs09 | CS 3600 | 88,3142 |
| cs10 | CS 3600 | 92,2213 |
| OC01 | OMNICAM | 86,1396 |
| OC02 | OMNICAM | 88,1285 |
| OC03 | OMNICAM | 88,1311 |
| OC04 | OMNICAM | 88,8929 |
| OC05 | OMNICAM | 87,8802 |
| OC06 | OMNICAM | 86,9021 |
| OC07 | OMNICAM | 88,525 |
| OC08 | OMNICAM | 85,8888 |
| OC09 | OMNICAM | 87,5482 |
| OC10 | OMNICAM | 87,6528 |
| TD01 | TRUE DEF | 93,0071 |
| TD02 | TRUE DEF | 94,8946 |
| TD03 | TRUE DEF | 85,152 |
| TD04 | TRUE DEF | 94,7277 |
| TD05 | TRUE DEF | 94,6059 |
| TD06 | TRUE DEF | 94,8761 |
| TD07 | TRUE DEF | 85,1771 |
| TD08 | TRUE DEF | 94,7895 |
| TD09 | TRUE DEF | 94,7211 |
| TD10 | TRUE DEF | 93,8812 |
| 3S01 | TRIOS 3 | 91,6998 |
| 3S02 | TRIOS 3 | 87,951 |
| 3S03 | TRIOS 3 | 91,7231 |
| 3S04 | TRIOS 3 | 92,6692 |
| 3S05 | TRIOS 3 | 88,2793 |
| 3S06 | TRIOS 3 | 91,52 |
| 3S07 | TRIOS 3 | 91,6112 |
| 3S08 | TRIOS 3 | 91,6225 |
| 3S09 | TRIOS 3 | 88,1415 |
| 3S10 | TRIOS 4 | 88,452 |
| ref01 | REFERENCE | 91,8766 |
| ref02 | REFERENCE | 91,8217 |
| ref03 | REFERENCE | 91,8366 |
| ref04 | REFERENCE | 87,083 |
| ref05 | REFERENCE | 87,1404 |

| TECHNIQUE | POINT 1 | POINT 2 | VECTOR1 | VECTOR2 | ANG1_2 | DIST1_2 |
| --- | --- | --- | --- | --- | --- | --- |
| Close Tray Technique | 0,0701 | 0,3035 | 5,6443 | 5,6755 | 10,6021 | 0,1429 |
| Close Tray Technique | 0,0446 | 0,2116 | 4,4023 | 4,8496 | 8,7034 | 0,1311 |
| Close Tray Technique | 0,0176 | 0,0384 | 0,4666 | 0,8169 | 0,7944 | -0,008 |
| Close Tray Technique | 0,0442 | 0,2095 | 4,3583 | 4,8011 | 8,6164 | 0,1298 |
| Close Tray Technique | 0,0119 | 0,0261 | 0,2687 | 0,5425 | 0,3619 | -0,0149 |
| Close Tray Technique | 0,0305 | 0,0367 | 0,591 | 0,9393 | 0,1724 | -0,0506 |
| Close Tray Technique | 0,0708 | 0,3065 | 5,7007 | 5,7323 | 10,7081 | 0,1443 |
| Close Tray Technique | 0,0178 | 0,0388 | 0,4713 | 0,8251 | 0,8023 | -0,0081 |
| Close Tray Technique | 0,0118 | 0,0258 | 0,2660 | 0,5371 | 0,3583 | -0,0148 |
| Close Tray Technique | 0,0308 | 0,0371 | 0,5969 | 0,9487 | 0,1741 | -0,0511 |
| Open Splinted | 0,0428 | 0,0785 | 0,7881 | 0,4802 | 0,261 | -0,1111 |
| Open Splinted | 0,0913 | 0,0913 | 1,2555 | 1,2713 | 0,5449 | -0,0479 |
| Open Splinted | 0,0638 | 0,1365 | 1,2244 | 0,7407 | 0,1084 | -0,047 |
| Open Splinted | 0,0587 | 0,0447 | 0,9993 | 0,5754 | -0,8383 | 0,0764 |
| Open Splinted | 0,0546 | 0,0853 | 0,3353 | 0,6942 | 0,76 | -0,0929 |
| Open Splinted | 0,0472 | 0,0237 | 0,5791 | 0,9549 | 0,3069 | -0,0595 |
| Open Splinted | 0,0912 | 0,0912 | 1,2541 | 1,2699 | 0,5443 | -0,0478 |
| Open Splinted | 0,0586 | 0,0447 | 0,9982 | 0,5748 | -0,8374 | 0,0763 |
| Open Splinted | 0,0434 | 0,0797 | 0,7999 | 0,4874 | 0,2649 | -0,1128 |
| Open Splinted | 0,0554 | 0,0866 | 0,3403 | 0,7046 | 0,7714 | -0,0943 |
| Open non-splinted | 0,0649 | 0,0841 | 2,0042 | 1,1428 | -0,6647 | -0,0119 |
| Open non-splinted | 0,0284 | 0,0184 | 0,8507 | 1,0739 | 1,1739 | -0,0315 |
| Open non-splinted | 0,0507 | 0,0424 | 0,5898 | 0,1135 | 0,4558 | 0,0926 |
| Open non-splinted | 0,0551 | 0,0774 | 1,0469 | 1,31 | 0,1541 | 0,0364 |
| Open non-splinted | 0,0374 | 0,016 | 0,4805 | 0,5945 | 0,2519 | -0,0189 |
| Open non-splinted | 0,0378 | 0,0162 | 0,4863 | 0,6016 | 0,2549 | -0,0191 |
| Open non-splinted | 0,0282 | 0,0183 | 0,8447 | 1,0664 | 1,1657 | -0,0313 |
| Open non-splinted | 0,0514 | 0,0430 | 0,5981 | 0,1151 | 0,4622 | 0,0939 |
| Open non-splinted | 0,0053 | 0,0074 | 0,1005 | 0,1258 | 0,0148 | 0,0035 |
| Open non-splinted | 0,0656 | 0,0850 | 2,0266 | 1,1556 | -0,6721 | -0,0120 |
| Master model | 0,0156 | 0,0249 | 0,2954 | 0,2125 | 0,0904 | 0,0215 |
| Master model | 0,0217 | 0,0145 | 0,3969 | 0,1861 | -0,1871 | -0,0284 |
| Carestream 3600 | 0,0103 | 0,0123 | 0,2797 | 0,1565 | -0,2475 | -0,0054 |
| Carestream 3600 | 0,0189 | 0,0075 | 0,3506 | 0,2574 | -0,1409 | -0,0108 |
| Carestream 3600 | 0,0123 | 0,0083 | 0,5895 | 0,2905 | -0,2478 | -0,0056 |
| Carestream 3600 | 0,0304 | 0,0266 | 0,5077 | 0,4612 | -0,2801 | -0,0015 |
| Carestream 3600 | 0,0065 | 0,0072 | 0,5776 | 0,2984 | -0,2814 | 0,0025 |
| Carestream 3600 | 0,024 | 0,0545 | 0,9103 | 0,4851 | -0,2636 | 0,0316 |
| Carestream 3600 | 0,0106 | 0,005 | 0,4764 | 0,1158 | -0,3506 | 0,0033 |
| Carestream 3600 | 0,0104 | 0,0217 | 0,3174 | 0,1983 | -0,3118 | 0,0166 |
| Carestream 3600 | 0,0073 | 0,0188 | 0,196 | 0,1734 | -0,078 | 0,0029 |
| Carestream 3600 | 0,0091 | 0,0325 | 0,2406 | 0,3047 | -0,2207 | 0,0277 |
| Carestream 3600 | 0,0091 | 0,0081 | 0,2098 | 0,0962 | -0,1395 | -0,0021 |
| Carestream 3600 | 0,0062 | 0,0201 | 0,1847 | 0,2464 | -0,1983 | 0,0176 |
| Carestream 3600 | 0,009 | 0,0167 | 0,0693 | 0,3621 | 0,2081 | 0,0188 |
| Carestream 3600 | 0,014 | 0,0186 | 0,1795 | 0,1279 | -0,026 | -0,0097 |
| Carestream 3600 | 0,0139 | 0,0174 | 0,2997 | 0,3611 | -0,3215 | 0,0086 |
| Carestream 3600 | 0,0058 | 0,0186 | 0,3774 | 0,205 | -0,141 | 0,0107 |
| Omnicam | 0,0398 | 0,0425 | 0,7761 | 0,4343 | -0,3483 | -0,0027 |
| Omnicam | 0,0341 | 0,0333 | 0,8489 | 0,3664 | -0,4667 | -0,0291 |
| Omnicam | 0,9592 | 0,0485 | 43,963 | 0,3622 | 41,6786 | 0,4023 |
| Omnicam | 0,0674 | 0,0797 | 0,0892 | 0,3253 | -0,2649 | -0,0351 |
| Omnicam | 0,0754 | 0,076 | 1,1179 | 0,5121 | -0,5002 | -0,0452 |
| Omnicam | 0,1382 | 0,1045 | 0,3658 | 0,433 | -0,2809 | -0,0346 |
| Omnicam | 0,1095 | 0,0779 | 1,1972 | 1,0143 | -0,1834 | -0,0282 |
| Omnicam | 0,2206 | 0,0549 | 0,8346 | 0,9231 | 0,012 | 0,0022 |
| Omnicam | 0,2865 | 0,1129 | 0,5503 | 0,6789 | -0,0193 | -0,0032 |
| Omnicam | 0,2159 | 0,0821 | 0,9692 | 0,9488 | -0,0206 | 0,0013 |
| Omnicam | 0,2979 | 0,1294 | 0,9903 | 0,9728 | -0,0205 | -0,0391 |
| Omnicam | 0,2597 | 0,1046 | 0,8924 | 0,5883 | -0,1715 | -0,0102 |
| TRIOS | 0,0251 | 0,0302 | 0,41 | 0,1616 | -0,1339 | -0,0027 |
| TRIOS | 0,0189 | 0,0075 | 0,3506 | 0,2574 | 0,1532 | 0,0003 |
| TRIOS | 0,0179 | 0,0187 | 0,3201 | 0,1651 | -0,1154 | -0,0099 |
| TRIOS | 0,017 | 0,0161 | 0,2718 | 0,0857 | -0,1084 | 0,0013 |
| TRIOS | 0,017 | 0,0161 | 0,2718 | 0,0857 | -0,1084 | 0,0013 |
| TRIOS | 0,0166 | 0,0255 | 0,5351 | 0,2503 | 0,047 | 0,0091 |
| TRIOS | 0,0175 | 0,0172 | 0,3797 | 0,161 | -0,2461 | 0,0004 |
| TRIOS | 0,0153 | 0,0232 | 0,2858 | 0,0945 | -0,0956 | -0,0044 |
| TRIOS | 0,0215 | 0,024 | 0,2267 | 0,1164 | -0,0746 | 0,0051 |
| TRIOS | 0,0269 | 0,0246 | 0,3626 | 0,1706 | -0,0829 | 0,0042 |
| TRIOS | 0,0189 | 0,0185 | 0,5753 | 0,1303 | -0,2492 | -0,0057 |
| TrueDefinition | 0,0398 | 0,0425 | 0,7756 | 0,4340 | -0,3481 | -0,0027 |
| TrueDefinition | 0,0345 | 0,0337 | 0,8582 | 0,3704 | -0,4718 | -0,0294 |
| TrueDefinition | 0,0689 | 0,0815 | 0,0912 | 0,3325 | -0,2708 | -0,0359 |
| TrueDefinition | 0,0254 | 0,0306 | 0,4150 | 0,1636 | -0,1355 | -0,0027 |
| TrueDefinition | 0,0345 | 0,0337 | 0,8584 | 0,3705 | -0,4719 | -0,0294 |
| TrueDefinition | 0,0398 | 0,0425 | 0,7761 | 0,4343 | -0,3483 | -0,0027 |
| TrueDefinition | 0,0341 | 0,0333 | 0,8489 | 0,3664 | -0,4667 | -0,0291 |
| TrueDefinition | 0,9592 | 0,0485 | 43,963 | 0,3622 | 41,6786 | 0,4023 |
| TrueDefinition | 0,0674 | 0,0797 | 0,0892 | 0,3253 | -0,2649 | -0,0351 |
| TrueDefinition | 0,0754 | 0,076 | 1,1179 | 0,5121 | -0,5002 | -0,0452 |

**One-Way ANOVA - PRECISION by SCANNERS FOR 2 IMPLANTS**

Dependent variable: PRECISION

Factor: METHOD

Number of observations: 155

Number of levels: 5

| 3S : Trios 3Shape |
| --- |
| C: Carestream |
| CI: Conventional Impresion |
| O: Omnicam Cerec |
| TD: True Definition 3M |

**The StatAdvisor**

This procedure performs a one-way analysis of variance for PRECISION. It constructs various tests and graphs to compare the mean values of PRECISION for the 5 different levels of METODO. The F-test in the ANOVA table will test whether there are any significant differences amongst the means. If there are, the Multiple Range Tests will tell you which means are significantly different from which others. If you are worried about the presence of outliers, choose the Kruskal-Wallis Test which compares medians instead of means. The various plots will help you judge the practical significance of the results, as well as allow you to look for possible violations of the assumptions underlying the analysis of variance.

**Summary Statistics for PRECISION**

| *METODO* | *Count* | *Average* | *Standard deviation* | *Coeff. of variation* | *Minimum* | *Maximum* | *Range* |
| --- | --- | --- | --- | --- | --- | --- | --- |
| 3S | 35 | 0,02944 | 0,0232687 | 79,0378% | 0,0045 | 0,0721 | 0,0676 |
| C | 42 | 0,0413143 | 0,0281204 | 68,0645% | 0,0052 | 0,0975 | 0,0923 |
| CI | 7 | 0,0722429 | 0,0568493 | 78,692% | 0,0171 | 0,1557 | 0,1386 |
| O | 43 | 0,0283256 | 0,0166173 | 58,6653% | 0,0058 | 0,0675 | 0,0617 |
| TD | 28 | 0,0271464 | 0,0117989 | 43,4637% | 0,0082 | 0,0485 | 0,0403 |
| Total | 155 | 0,0338671 | 0,0256711 | 75,7994% | 0,0045 | 0,1557 | 0,1512 |

| *METODO* | *Stnd. skewness* | *Stnd. kurtosis* |
| --- | --- | --- |
| 3S | 1,31382 | -1,8218 |
| C | 1,55943 | -1,14513 |
| CI | 0,575728 | -1,05325 |
| O | 1,94022 | -0,146892 |
| TD | 0,987121 | -0,777798 |
| Total | 8,18427 | 9,19071 |

This table shows various statistics for PRECISION for each of the 5 levels of METODO. The one-way analysis of variance is primarily intended to compare the means of the different levels, listed here under the Average column. Select Means Plot from the list of Graphical Options to display the means graphically.

WARNING: There is more than a 3 to 1 difference between the smallest standard deviation and the largest. This may cause problems since the analysis of variance assumes that the standard deviations at all levels are equal. Select Variance Check from the list of Tabular Options to run a formal statistical test for differences among the sigmas. You may want to consider transforming the values of PRECISION to remove any dependence of the standard deviation on the mean.

**ANOVA Table for PRECISION by METODO**

| *Source* | *Sum of Squares* | *Df* | *Mean Square* | *F-Ratio* | *P-Value* |
| --- | --- | --- | --- | --- | --- |
| Between groups | 0,0159094 | 4 | 0,00397734 | 6,97 | 0,0000 |
| Within groups | 0,0855772 | 150 | 0,000570515 |  |  |
| Total (Corr.) | 0,101487 | 154 |  |  |  |

The ANOVA table decomposes the variance of PRECISION into two components: a between-group component and a within-group component. The F-ratio, which in this case equals 6,9715, is a ratio of the between-group estimate to the within-group estimate. Since the P-value of the F-test is less than 0,05, there is a statistically significant difference between the mean PRECISION from one level of METODO to another at the 95,0% confidence level. To determine which means are significantly different from which others, select Multiple Range Tests from the list of Tabular Options.

**Kruskal-Wallis Test for PRECISION by SCANNER**

| *SCANNER* | *Sample Size* | *Average Rank* |
| --- | --- | --- |
| 3S | 35 | 67,2714 |
| C | 42 | 90,131 |
| CI | 7 | 111,714 |
| O | 43 | 72,6512 |
| TD | 28 | 73,0 |

Test statistic = 9,97346 P-Value = 0,0408771

The Kruskal-Wallis test tests the null hypothesis that the medians of PRECISION within each of the 5 levels of SCANNER are the same. The data from all the levels is first combined and ranked from smallest to largest. The average rank is then computed for the data at each level. Since the P-value is less than 0,05, there is a statistically significant difference amongst the medians at the 95,0% confidence level. To determine which medians are significantly different from which others, select Box-and-Whisker Plot from the list of Graphical Options and select the median notch option.

**Multiple Range Tests for PRECISION by METODO**

Method: 95,0 percent LSD

| *METODO* | *Count* | *Mean* | *Homogeneous Groups* |
| --- | --- | --- | --- |
| TD | 28 | 0,0271464 | X |
| O | 43 | 0,0283256 | X |
| 3S | 35 | 0,02944 | X |
| C | 42 | 0,0413143 | X |
| CI | 7 | 0,0722429 | X |

| *Contrast* | *Sig.* | *Difference* | *+/- Limits* |
| --- | --- | --- | --- |
| 3S - C | * | -0,0118743 | 0,0108016 |
| 3S - CI | * | -0,0428029 | 0,0195408 |
| 3S - O |  | 0,00111442 | 0,0107443 |
| 3S - TD |  | 0,00229357 | 0,0119662 |
| C - CI | * | -0,0309286 | 0,0192675 |
| C - O | * | 0,0129887 | 0,0102388 |
| C - TD | * | 0,0141679 | 0,0115145 |
| CI - O | * | 0,0439173 | 0,0192354 |
| CI - TD | * | 0,0450964 | 0,0199437 |
| O - TD |  | 0,00117915 | 0,0114608 |

* denotes a statistically significant difference.

**The StatAdvisor**

This table applies a multiple comparison procedure to determine which means are significantly different from which others. The bottom half of the output shows the estimated difference between each pair of means. An asterisk has been placed next to 7 pairs, indicating that these pairs show statistically significant differences at the 95,0% confidence level. At the top of the page, 3 homogenous groups are identified using columns of X's. Within each column, the levels containing X's form a group of means within which there are no statistically significant differences. The method currently being used to discriminate among the means is Fisher's least significant difference (LSD) procedure. With this method, there is a 5,0% risk of calling each pair of means significantly different when the actual difference equals 0.

**Mood's Median Test for PRECISION by SCANNER**

Total n = 155

Grand median = 0,0278

| *SCANNER* | *Sample Size* | *n<=* | *n>* | *Median* | *95,0% lower CL* | *95,0% upper CL* |
| --- | --- | --- | --- | --- | --- | --- |
| 3S | 35 | 21 | 14 | 0,0159 | 0,0107372 | 0,0499628 |
| C | 42 | 17 | 25 | 0,03315 | 0,0226436 | 0,0507693 |
| CI | 7 | 2 | 5 | 0,0369 |  |  |
| O | 43 | 21 | 22 | 0,0279 | 0,0187607 | 0,0330785 |
| TD | 28 | 17 | 11 | 0,02685 | 0,0203668 | 0,0299387 |

Test statistic = 5,51227 P-Value = 0,238653

**The StatAdvisor**

Mood's median test tests the hypothesis that the medians of all 5 samples are equal. It does so by counting the number of observations in each sample on either side of the grand median, which equals 0,0278. Since the P-value for the chi-square test is greater than or equal to 0,05, the medians of the samples are not significantly different at the 95,0% confidence level. Also included (if available) are 95,0% confidence intervals for each median based on the order statistics of each sample.
